# Using a qualitative approach for understanding hospital-affiliated integrated clinical and fitness facilities: characteristics and members’ experiences

**DOI:** 10.1186/s12889-015-1934-8

**Published:** 2015-06-19

**Authors:** Jingzhen Yang, Diana Kingsbury, Matthew Nichols, Kristin Grimm, Kele Ding, Jeffrey Hallam

**Affiliations:** The Department of Social and Behavioral Sciences, Kent State University, Kent, OH USA; The Research Institute at Nationwide Children’s Hospital, The Ohio State University, Columbus, OH USA; The Department of Health Sciences, Kent State University, 142 Nixson Hall, Kent, OH 44242 USA

**Keywords:** Integrated facility, Clinical and fitness services, Wellness promotion

## Abstract

**Background:**

With health care shifting away from the traditional sick care model, many hospitals are integrating fitness facilities and programs into their clinical services in order to support health promotion and disease prevention at the community level. Through a series of focus groups, the present study assessed characteristics of hospital-affiliated integrated facilities located in Northeast Ohio, United States and members’ experiences with respect to these facilities.

**Methods:**

Adult members were invited to participate in a focus group using a recruitment flyer. A total of 6 focus groups were conducted in 2013, each lasting one hour, ranging from 5 to 12 participants per group. The responses and discussions were recorded and transcribed verbatim, then analyzed independently by research team members. Major themes were identified after consensus was reached.

**Results:**

The participants’ average age was 57, with 56.8 % currently under a doctor’s care. Four major themes associated with integrated facilities and members’ experiences emerged across the six focus groups: 1) facility/program, 2) social atmosphere, 3) provider, and 4) member. Within each theme, several sub-themes were also identified. A key feature of integrated facilities is the availability of clinical and fitness services “under one roof”. Many participants remarked that they initially attended physical therapy, becoming members of the fitness facility afterwards, or vice versa. The participants had favorable views of and experiences with the superior physical environment and atmosphere, personal attention, tailored programs, and knowledgeable, friendly, and attentive staff. In particular, participants favored the emphasis on preventive care and the promotion of holistic health and wellness.

**Conclusions:**

These results support the integration of wellness promotion and programming with traditional medical care and call for the further evaluation of such a model with regard to participants’ health outcomes.

## Background

With recent reforms to the American health care system, the prevention of disease, rather than the treatment of disease, is receiving increased attention [1]. There are growing opportunities for health care providers and health systems to be actively involved in health promotion through physical activity among patients and communities [2]. An example of such an initiative is Exercise is Medicine®, a nonprofit campaign launched in 2007 by the American College of Sports Medicine and the American Medical Association [3]. Exercise is Medicine® advocates that all health care providers include recommendations for physical activity as a standard component of medical treatment. Such recommendations include checking physical activity levels as a vital sign in every patient visit, linking patients with health and fitness professionals, and even prescribing physical activity within the treatment regimen [3].

To facilitate such a paradigm shift, many hospitals and their respective health systems are expanding their reach by incorporating an approach that goes beyond traditional sick care to integrating fitness facilities and programs within their clinical services [4–6]. The number of such integrated facilities has increased nationwide, growing from roughly 79 facilities in 1985 to more than 1000 in 2010 [7]. These integrated facilities include clinical and fitness services and programs that aid in the improvement of both individual and community health and wellness, thereby reducing health care costs [8].

As the first of such integrated facilities in Northeast Ohio, United States, Akron General LifeStyles provides clinical services (i.e., physical and occupational therapy), physician offices (i.e., primary care), retail health care services (i.e., pharmacies), and fitness centers (i.e., cardio/strength equipment, group fitness space, therapy, and lap pools) at three locations [9]. Akron General LifeStyles also employs many health professionals, including but not limited to: therapists, physicians, pharmacists, fitness or group exercise instructors, personal trainers, nutrition counselors, and lifeguards. Aiming to reduce risk factors for disease, support patient recovery, and improve members’ health and wellness, Akron General LifeStyles offers convenient hours of operation, state-of-the-art equipment, trained staff, spacious exercise areas, and health and wellness services tailored to individuals of all ages [10]. While such hospital-affiliated integrated facilities represent an opportunity to promote health and well-being within the health care system and community, research on what services these integrated facilities offer and how these services may promote health and wellness within the community is lacking [8].

Through a series of focus groups, the present study assessed characteristics of the hospital-affiliated integrated facilities located in Northeast Ohio, United States and members’ experiences with these facilities. The information obtained from this study may be helpful in future impact evaluations of integrated models as a new paradigm of health promotion and disease prevention.

## Methods

### Study participants

Participants were eligible to participate if they were 18 years of age or older, English-speaking, and current members of one of the three Akron General LifeStyles facilities.

### Study procedure

Following approval of the study and consent process by both Kent State University and Akron General Institutional Review Boards, the study was introduced to members through a flyer that included brief descriptions of the study and also the email and phone number of the research assistant (MN), who was a Master of Public Health candidate. The flyer was published in a newsletter and while also posted at each of the three LifeStyles facilities. With assistance from the staff, the study sign-up sheet was provided at the three study sites to those who expressed interest in participating. The research assistant (MN) contacted a convenience sample of individuals who signed up for the focus group, screened for eligibility, and scheduled dates and times of the focus groups via email or phone, based upon the availability of the potential participants. This study also included participants who are current members of a voluntary, non-elected, advisory board (the purpose of which is to provide member insight to the facility), each of whom held a one-year, non-renewable term.

### Data collection

Six focus groups were conducted on-site at each of the three facilities (two focus groups per location) between October and December of 2013. The focus groups were convened on weekday afternoons or evenings, typically before or after the participants’ regularly scheduled visits to the center.

At the beginning of each focus group, the researchers introduced the study and consent process. Focus group participants were given the opportunity to ask questions regarding the study and consent process before they signed the consent form. Following consent, each focus group discussion was moderated by two trained research team members (MN, JY, DK, KD) all of whom were either faculty or students of Kent State University. Each session lasted roughly 60 min and was audio recoded via two digital recorders in order to ensure that all comments were audible for review. At the end of the focus group, each participant was asked to complete a short questionnaire regarding his/her demographics (e.g., age, gender, race, education, employment, and current health status) as well as frequency of visits to the facility and use of programs offered by LifeStyles (Table 1).

**Table 1:** Characteristics of Participants (*n* = 45)

		n	%
Gender	Female	27	58.7
	Male	19	41.3
Race	White	44	95.7
	Other race	2	4.4
Education	College graduate or higher	28	60.9
	Some college or lower	18	39.1
Employment	Full time	19	41.3
	Part time	5	10.9
	Self-employed	2	4.4
	Retired	18	39.1
	Unemployed	2	4.4
Frequency of visit to LifeStyles	5-7 days/week	21	48.8
	3-4 days/week	19	44.2
	1-2 days/week	3	7.0
Used other fitness facilities previously	Yes	33	73.3
	No	12	26.7
Currently under a doctor’s care	Yes	25	56.8
	No	19	43.2
Self-rated overall health	Poor	2	4.4
	Average	27	58.7
	Good	17	37.0
Age (Mean, Median, Std)		57.2, 58.0,12.4	
Distance to facility/miles (Mean, Median, Std)		6.0, 5.0, 5.0

**Table 2:** Integrated Clinical and fitness facility focus group: themes, subthemes, and quotes

Theme	Subtheme	Quote
Facility/Program		
	Physical Environment	
		*“…working out on those machines you feel like you have room, you’re not on top of the other person working out so there's a lot of privacy and good space.’*
	Superiority	
		*“[other gym], I have a free membership there. I won’t go; I would rather pay and come here because you get so much more”.*
		*“[…] but I don’t feel comfortable in [other gym], you know you got trainers in there, but it’s just not a professional atmosphere like here”.*
		*“Yeah, we compared the [other gym] to this and we have been, I have been going on and off to different places to exercise since I was about 30 I think and so I’ve been to different places […] I’ll tell you what made us decide not to go to [other gym] was the crowdedness, and they were a lot less money to go there, they were less money but then when we came over here we said ’Oh no, this is where we’re gonna go”.*
	Convenience	
		*“…they have lots of exercise classes you can come to and because I’m so busy, if I miss a class I know I can easily get to another class or I can just hop on a treadmill”.*
		*“We live within 2 miles of here, it’s very convenient”.*
	Programs	
		*“Every time I’m here, [trainer] has customized a program that is targeted towards what I want to work on”.*
		*“[T]hat’s one of the things that was attractive to me is the idea that there would be a variety”.*
Social Atmosphere		
	Low Pressure for Cosmetics	
		*“…you don’t feel like people are judging you for what you are doing”.*
		*“…you don’t have to get dressed up to come here which is nice. I mean, it’s not like you know, there’s lots of spandex or anything, you know you feel ok just kind of shlumping in, you know in middle age”.*
		*“I like that it’s an older crowd, it’s not all young beautiful people. There are normal people here”.*
	Making New Friends	
		*“It’s a social thing besides fitness too. I retired, I was working in Florida and when I retired, I moved up here. I’ve met 40 or 50 people since I’ve been here, it’s amazing”.*
		*“We know each other; we share each other’s sorrows, joys, and stuff”.*
		*“Being alone, I have met a lot of nice people here that I have become friends with and so there’s a social aspect to all this and I find that very rewarding”.*
	Social Support	
		*“I signed up here with a friend and my friend doesn’t come anymore, but I don’t need my friend to help come, I have the friends here that help.*
		*“You’ve got all that community support picking you up and moving you”.*
		*“We know each other, we share each other’s sorrows, joys, and stuff. Somebody needs help and we offer our suggestion, you know it’s not anything great but we consider ourselves a family”.*
Provider		
	Knowledgeable	
		*“I like the fact that they have professionals you can ask about your exercise program”.*
		*“I think the medical model shows through like they will work with you around some issues. Like, I’ve been having some knee issues and I’m getting some injections for it so you know they’ve modified my exercises in order to be able to accommodate that but still get a decent work out”.*
	Friendly	
		*“I like the staff and the friendliness of the, the overall feel of the place better than some other places I’ve gone to”.*
		*“[…] but it is nice, you walk in and you’re always greeted by a warm, friendly smile, just everyone is very warm and friendly, to me, to my kids, to everyone. I think if it wasn’t like that, I wouldn’t come back”.*
		*“The staff, you know, it’s like Cheers, everybody knows your name”.*
	Attentive	
		*“I feel like everyone has an interest in me, I’m sure you guys feel like that too, but I feel they care about me as a person”.*
		*“There’s people here when you come in and you can ask them questions. I mean the people are here to help you and they will stop and answer your questions and do everything to make you comfortable”.*
		*“…she went above and beyond and she stayed with us and she walked us down to the emergency room and she made sure that we were settled and checked in before she left”.*
Member		
	Sense of Accomplishment	
		*“Yeah, and then a month later I did my first run in my entire life […]”*
		*“I get up in the morning, have a coffee and look forward to getting dressed, coat and stuff and head out here. It just became a routine, they call the place Lifestyles. It is a change and people said you really go to the gym that often? Yes”.*
		*“I joined in 2008 because my husband needed to join and he never came and I did (laughs). From 2008, when I had my first assessment, I hadn’t had an assessment for five years. When I had my second assessment five years later, from being almost 60 to being 65, my flexibility has improved and I say thank you LifeStyles”.*
	Motivation to Join	
		*“I feel like the members here are here because they want to be healthy, not because they are trying to pick up someone, it’s everybody is here for a common good and overall health”.*
		*“And I’m with (name removed), I want to keep my health, I’ve got arthritis, and I want to keep my health, and I don’t want to have to live with my daughter (laughs)”.*
		*“I told my children I never wanted to live with them so this is my part-time job. I said I would make it my part-time job by coming here and staying healthy, that’s my goal in life…”*

### Focus group guide

The semi-structured focus group interview guide was developed through the input of LifeStyles directors, the program and service materials, and current literature on the role of health care systems in promoting physical activity [7–9]. It was reviewed by both university researchers and hospital practitioners prior to implementation and was modified following the initial focus group. The guide facilitated the overall progression of the focus groups without hindering the participant’s ability to respond to questions or requiring the moderator to read verbatim. The interview guide was comprised of four key, open-ended questions to provide insight on participants’ immediate experiences as a member of the integrated facility. Specifically, these included: 1) the characteristics of the integrated facility that have influenced participants’ decisions to initiate and maintain membership, 2) participants’ views regarding the integrated facility in general, 3) participants’ opinions of current services and programs provided by the integrated facility, and 4) suggestions for future improvements. Accompanying each question was a set of probing questions to stimulate additional responses. Each session continued to build upon the next as common themes were identified across groups.

### Data analysis

After each focus group, audio files from the session were transcribed verbatim by a member of the research team (KG). Copies of the transcripts were provided to each of the four members of the research team (JY, DK, MN) to be analyzed. The copies of the transcripts were compared with the field notes and evaluated independently, identifying common themes across the focus groups. Only themes consistent across the focus groups were selected for the initial analysis. As additional data were analyzed, the coding process continued until no new themes emerged. When dominant themes were identified, consensus was reached among the research team. The resulting themes were then paired with direct quotations from the transcribed audio. Additionally, the researchers identified sub-themes that were consistently shared between the focus groups.

## Results

### Characteristics of participants

Each focus group had 5-12 participants (*n* = 46). Of the 46 participants, 58.7 % were female, 95.6 % were white, 60.9 % received a college or post-graduate degree, and 39.1 % were currently retired. The participants’ average age was 57 (SD = 12.4), with 56.8 % currently under a doctor’s care. The average distance a member traveled to their center was 6 miles (SD = 5.0), and the average length of membership was 3.4 years, with membership length ranging from 1 month to 16 years.

Nearly three-quarters (73.3 %) of the participants had used other facilities prior to acquiring LifeStyles membership, and 86.7 % of participants were currently using the LifeStyles facility exclusively. The average exercise duration was 1.2 h, with 48.8 % of participants exercising 5-7 days per week. On average, the participants rated their satisfaction with the LifeStyles facility as a 4.8 on a 5.0 scale, indicating that members were very satisfied with their experience.

### Four major themes

Four major themes associated with integrated facilities and members’ experiences emerged across the six focus groups: 1) facility/program, 2) social atmosphere, 3) provider, and 4) member (Table 2). We excluded three less common themes in this study because these were specific to one of the three sites while not relevant to the other two (i.e., facilities support family involvement, need for weight management programs, and need for community education and membership). The results presented below are organized by theme and accompanying sub-themes.

### Facility/program

Commonalities across the focus groups were seen in the overall positive views of members in their experience with their respective facilities. Comments regarding the facility included those directly related to the physical environment, as well as to the programs of which the facility offers. Results indicate that members had an overall favorable participatory experience with the integration of clinical services and a fitness facility. The sub-themes identified were physical environment, superiority, convenience, programs, and integration.

#### Physical environment

Respondents across all three locations presented favorable remarks regarding cleanliness, spaciousness, and the noise level of the facilities. Respondents also noted that LifeStyles was less “chaotic”, with more room to exercise and use equipment when compared to other fitness facilities. One participant compared LifeStyles with other facilities he had used:

*“…working out on those machines you feel like you have room, you’re not on top of the other person working out so there’s a lot of privacy and good space”.*

Another participant noted her perception that the facility was “cleaner” due to the presence of medical services housed within the facility:

*“…it’s cleaner because of the fact that the hospital and emergency room, and all these facilities are here, so therefore I think that helps keep it cleaner”.*

#### Superiority

Respondents also indicated they had better experiences with LifeStyles as compared to other fitness facilities and, since becoming members, stated that they would “never go back”. The members believed that the program offerings, amenities, and physical aesthetics associated with LifeStyles made membership more worthwhile as compared to other facilities. Some members even preferred it over gyms with free membership or those covered by their insurance plans. One individual stated:

*“[other gym], I have free membership there. I won’t go; I would rather pay and come here because you get so much more”.*

Another participant remarked:

*“Yeah, we compared the [other gym] to this and we have been, I have been going on and off to different places to exercise since I was about 30 I think and so I’ve been to different places […] I’ll tell you what made us decide not to go to [other gym] was the crowdedness, and they were a lot less money to go there, they were less money but then when we came over here we said ‘Oh no, this is where we’re gonna go’”.*

#### Convenience

The majority of respondents noted that the locations of the facilities were convenient and close to where they lived and/or worked. Members could visit a different LifeStyles location as needed. The group exercise class schedule was flexible, with classes available during multiple times of the day, allowing classes to fit into members’ personal schedules. One participant stated:

*“…they have lots of exercise classes you can come to and because I’m so busy, if I miss a class I know I can easily get to another class or I can just hop on a treadmill”.*

#### Programs

Frequently mentioned across focus group discussions was how LifeStyles consistently maintained effective client-centered programs, which enhanced members’ experiences while simultaneously helping them reach their goals. Participants often commented that the LifeStyles staff presented them with customized fitness plans, tailored to their specific needs, goals, and limitations. The variety of classes and programs offered members the opportunity to choose programs and classes based on their interests and needs. One participant remarked:

“*One of the things I like about it is that the trainer understands what the goals are…it’s very clear [name of staff omitted] knows what I want to work on and how hard or bad I am willing to work to get there. Every time I’m here, he has customized a program that is targeted towards what I have to work on. So I like that, it doesn’t feel like he’s winging it, it feels like he’s putting the effort in and I’m putting effort in and getting results”.*

#### Integration

A key feature of LifeStyles is the availability of clinical and fitness services offered “under one roof”. Several participants commented that they initially attended physical therapy, becoming members of the fitness facility afterwards. Many participants noted that after becoming members, they began using the clinical services in the facility. As such, the ease of transition between the available clinical and fitness services was an important consideration in their choice to become members. With integrated services “under one roof,” respondents felt they did not have to go far to have their needs met.

*“It’s a full service center […]. We go and we get our blood work done here, […]. When you’re comfortable in a place, that comfort travels through to the other things that they are offering here”.*

Some participants also noted that exercising in a facility that included an emergency room made them feel safer during their workouts.

*“I feel a little bit safer in my workouts knowing that there’s an emergency room right there”.*

With clinical services in the same location as the wellness center, many felt the dichotomy brought the concept of preventive care to the forefront, offering a holistic approach to the maintenance and promotion of good health.

### Social atmosphere

Many participants expressed that the LifeStyles social environment played an important role in their maintenance of a long-term membership and keeping physically active. Specifically, the accepting environment and the social support gained from the development of new friendships created an atmosphere where individuals were motivated to keep returning.

#### Low pressure for cosmetics

Many focus group participants felt there was little to no pressure from their peers and/or the facility for cosmetics, the need to dress up, or to impress others when coming to the center. Many felt comfortable being around others their own age that no longer had a “youthful” body shape. One participant said:

*“I like that it’s an older crowd, it’s not all young beautiful people. There are normal people here”.*

Another participant noted that she believed that people feel comfortable because:

“*…you don’t feel like people are judging you for what you are doing.*”

One participant also indicated that:

*“You don’t have to get dressed up to come here which is nice. I mean it’s not like, you know, there’s lots of spandex or anything, you know, you feel ok just kind of shlumping in, you know in middle age”.*

#### Making new friends / social support

Many participants commented on the importance of making friends within the facility as a motivator for continued membership. Social support was identified as an important aspect of membership across focus groups.

*“Being alone, I have met a lot of nice people here that I have become friends with and so there’s a social aspect to all this and I find that very rewarding”.*

“*I signed up here with a friend and my friend doesn’t come anymore, but I don’t need my friend to help me come, I have the friends here that help.*”

Many participants experienced a family-like atmosphere, sharing coffee together, taking turns bringing treats to class, and serving as a source of social support for each other.

“*We know each other; we share each other’s sorrows, joys, and stuff. Somebody needs help and we offer our suggestion, you know it’s not anything great but we consider ourselves a family”.*

### Provider

Consistent across focus group discussions was the undertone of superiority of the services provided by LifeStyles as compared to other facilities, and more specifically, the staff who was directly involved with making their experience positive.

#### Knowledgeable

The LifeStyles wellness staff was regarded as well-educated, professional, and knowledgeable. Additionally, they were successful in disseminating health and exercise information and guidance when requested, individualizing their recommendations to meet the needs of the member.

*“I like the fact that they have professionals you can ask about your exercise program”.*

#### Friendly

Many respondents indicated that the staff was warm, courteous, and respectful while also noting that they acquired close, caring relationships with the members more reminiscent of friendships than of traditional patient/provider relationships.

*“The staff, you know, it’s like Cheers, everybody knows your name”.*

#### Attentive

The staff was consistently praised for the extent of their service to clients. They were attentive to the needs of each participant and often went above and beyond the scope of duty.

*“…she went above and beyond and she stayed with us and she walked us down to the emergency room and she made sure that we were settled and checked in before she left”.*

### Member

In contrast to most fitness facilities, members were driven by health and wellness goals rather than aesthetic motivators, such as improvements in health and independence.

#### Sense of accomplishment

Many members surpassed fitness and/or health goals as a result of using their LifeStyles membership, regularly identifying these accomplishments through built-in program assessments.

*“I joined in 2008 because my husband needed to join and he never came and I did (laughs). From 2008, when I had my first assessment, I hadn’t had an assessment for five years. When I had my second assessment five years later, from being almost 60 to being 65, my flexibility has improved and I say thank you LifeStyles”.*

Another member added:

*“Yeah, and then a month later I did my first run in my entire life […]”*

#### Motivation to join

Many participants indicated their motivation to join was facilitated by health-related goals instead of aesthetic ones.

*“I feel like the members here are here because they want to be healthy, not because they are trying to pick up someone, it’s everybody is here for a common good and overall health”.*

In addition, goals of health improvement and living independently motivated many participants to take control of their health, treating health and wellness as a regular component of their day. One woman said:

*“I told my children I never wanted to live with them so this is my part-time job. I said I would make it my part-time job by coming here and staying healthy, that’s my goal in life…”*

Another participant added:

*“And I’m with (name removed), I want to keep my health, I’ve got arthritis, and I want to keep my health, and I don’t want to have to live with my daughter (laughs)”.*

## Discussion

This is one of few studies that describe the characteristics of integrated clinical and fitness facilities and members’ experiences. The results indicate that participants had favorable views of and experiences with the integration of clinical and fitness services. In particular, participants reported it was the integrated design of the facilities that influenced their membership, either through an introduction to the fitness facility via their use of clinical services, or by choosing the facility for clinical services because of their exposure through the fitness programs. The seamless connection between medical care and the promotion of wellness and active lifestyles through the fitness facility was one of the characteristics that participants appreciated most.

Facilities such as LifeStyles provide the infrastructure to deliver integrated services that simultaneously provide clinical care and total wellness programming in ways not previously seen in traditional fitness facilities [11]. The inclusion of such services in community-based facilities represents an opportunity for the health care system to better respond to community health needs as well as improve the quality of the health care process. This ensures that patient health is addressed holistically – a departure from the traditional “sick care” model that has characterized the U.S. health care system [1]. With the implementation of the Patient Protection and Affordable Care Act, the emphasis on health has begun to shift from disease treatment and “sick care” to prevention [7]. While this shift may have been years in the making, there is an opportunity for the health care system to be more actively involved in prevention efforts at the community level [1]. The results of this study have important implications for how the health care system can play a role in promoting health and wellness through integrated facilities.

In the present study, participants were, on average, around 57 years old, and more than half reported they were currently under a doctor’s care. This older adult population represents an opportunity to consider how an integrated facility may not only address the health needs of the aging, but how it may also respond to the health needs of individuals living with chronic diseases that require continued care (i.e., diabetes, heart disease). As it currently stands, billions of dollars are spent each year to treat and respond to chronic diseases in the U.S., with additional costs associated with the health risk behaviors that lead to many chronic diseases [12]. It is possible that through the presence of integrated health facilities at the community level, greater savings to the health care system may be realized by providing the means necessary for individuals to prevent disease before it occurs.

The holistic approach of the LifeStyles facilities offers members a continuum of care, transitioning between acute services, chronic services, pharmacy, wellness activities, and other integrated options, all of which facilitate quick, convenient access [13]. A recent study showed that in the last two decades, clinically-based integrated facilities have increased by almost 1000 % [14]. Fundamentally, the aim of integrated facilities is to have all necessary services conveniently located in one setting in order to reduce travel time to specialists and eliminate out-of-system referral issues [13]. LifeStyles members can access services before and after their scheduled wellness classes and/or workout times, easing the stress commonly associated with traditional outpatient care. Future programs may offer off-site services to a local community in order to engage more potential members.

Consistent with previous findings [15], this study also found that driving forces for participation in clinically-based wellness programs include: convenience, accessibility, enjoyment, inclusion of a social component, and an on-hand, knowledgeable staff. In addition, many participants used the integrated facility as a source of social support, finding comfort with others their age that no longer had a “youthful” body shape or had the same health conditions as themselves. Additional studies are necessary to quantify the physical and social benefits of such integrated facilities and the significance of these benefits in members’ decisions to keep physically active.

The study findings must be interpreted in light of several limitations. First, participants of this study consisted of active LifeStyles members. Therefore, the opinions and experiences of former members and non-active members were not assessed in this study, lending the inability to determine whether these findings apply equally to current, non-active, and/or former members. In addition, the convenience sampling method used can lead to selection bias which may skew results as those willing to participate may be more likely to be satisfied with the facility. Convenience sampling also precluded the ability to calculate response rates. Secondly, some of the participants were current advisory committee members, so their experiences with the facility and the barriers to participation may differ from those of a more traditional member. However, we were not able to assess these differences as we were unable to identify those individuals in our recordings. It should be noted that due to the inability to identify individuals in our recordings, quotations used to represent the members’ perceptions were not able to be identified by a participant number. Third, participants were encouraged to disclose their perceptions and opinions regarding the discussion topics, but as with all focus groups, a level of social desirability may have been present. Finally, saturation may have not been achieved through the six focus groups conducted, as additional focus groups may have produced additional themes.

## Conclusions

In addition to providing an opportunity to improve health care and encourage active living, integrated clinical and fitness facilities offer potential for cost savings to both participants and health care systems. Additional research should further determine the acceptability of such facilities as well as their impact on health and wellness of the participants. The results of this study support hospital-affiliated integrated clinical and fitness models as a new paradigm of health promotion and disease prevention.
